# Postural instability and lower extremity dysfunction in upper motor neuron-dominant amyotrophic lateral sclerosis

**DOI:** 10.3389/fneur.2024.1406109

**Published:** 2024-07-15

**Authors:** Xiangyi Liu, Lu Chen, Shan Ye, Xiaoxuan Liu, Yingshuang Zhang, Dongsheng Fan

**Affiliations:** ^1^Department of Neurology, Peking University Third Hospital, Beijing, China; ^2^Beijing Municipal Key Laboratory of Biomarker and Translational Research in Neurodegenerative Diseases, Beijing, China; ^3^Key Laboratory for Neuroscience, National Health Commission, Ministry of Education, Peking University, Beijing, China

**Keywords:** amyotrophic lateral sclerosis, upper motor neuron, gait instability, lower extremity, cohort study

## Abstract

**Background:**

Upper motor neuron-dominant ALS (UMND ALS) is recognized to have early onset and good prognosis, but may have a rapid decline in motor function due to gait instability in the early stage. We investigated changes in lower extremity function in UMND ALS, particularly UMND ALS patients accompanied with postural instability or repeated falls (UMND ALS plus).

**Results:**

Among the 2,353 ALS patients reviewed, 211 (9.0%) had UMND ALS. UMND ALS had a longer diagnosis delay and restricted symptoms. Although UMND ALS patients had better lower extremity function and strength than matched classic ALS patients on first evaluation, there was no difference in the time of needing assistance or not being able to walk after disease onset. In contrast, UMND ALS plus has severe UMN symptoms and a more rapid decline in motor function. The lower extremity function was no better than that in the matched classic ALS. The prognosis of UMND ALS and UMND ALS plus were significantly better than those of overall ALS.

**Conclusion:**

UMND ALS has restricted symptoms but has a rapid decline in lower extremity function in the early stage of the disease. The motor function decline of UMND ALS plus is as fast as classic ALS. Whether these patients represent a distinct subgroup of ALS deserves further investigation.

## Introduction

Amyotrophic lateral sclerosis (ALS) is a neurodegenerative disease characterized by degeneration of upper and lower motor neurons (1). The clinical picture of ALS is heterogeneous, including classic ALS, which is characterized by widespread muscle atrophy and corticospinal tract involvement, and flail arm syndrome, which is dominated by restricted muscle atrophy in both upper extremities. In addition, upper motor neuron-dominant ALS (UMND ALS) mainly involves upper motor neurons (UMNs), with only minor lower motor neuron (LMN) lesions (2, 3). Although these clinical phenotypes share similar pathological hallmarks, the prognosis is quite different, which suggests heterogeneity in ALS (4).

The prevalence of UMND ALS ranges from 6.5 to 22.2% among all ALS patients (5, 6). UMND ALS is generally considered to have an earlier disease onset, a better prognosis and a slower rate of progression than classic ALS (7). Previous studies have suggested that its better prognosis may be related to preservation of respiratory function (6). The subtle LMN damages differentiate UMND ALS from primary lateral sclerosis (PLS), which has a better prognosis. Despite the apparent differences, these three phenotypes (ALS, UMND ALS and PLS) still belong to a continuous spectrum of disorder (8).

Although the prognosis of UMND ALS is better than other ALS phenotypes, the severe spasticity caused by upper motor neuron damage has a significant impact on the activity of daily living, especially lower limb function and ambulatory abilities. Some studies also revealed that UMND ALS and PLS patients have gait instability and frequent falls in the early disease stage (9–11). These manifestations predominantly affect lower extremity function and cause the revised ALS functional rating scale (ALSFRS-R) score, especially the lower extremity part, to significantly decrease in the first few years of the disease.

The pathophysiological mechanism of postural instability in ALS patients is still not fully revealed. UMN lesions were suggested to be one of the major reasons for postural instability, but recent studies also indicate that frontal lobe dysfunction (premotor area, precentral gyrus, parietal lobule, dorsolateral prefrontal) also has an impact on motor dysfunction (12, 13). Some of these patients even have parkinsonism features such as bradykinesia and tremor (ALS-plus) (12, 14–17). There is no conclusion that postural instability represents a distinct feature of a subgroup of ALS patients rather than the consequence of more severe UMN signs in the lower limbs.

In this study, we describe the clinical features and the phenomenon of mismatch between the rate of lower extremity function decline and prognosis in patients with UMND ALS in a large ALS cohort. We compared these patients with matched classical ALS patients and discussed the early decline in motor function and the difference between UMND ALS with and without postural instability.

## Methods

### Study design and participants

We collected data from patients who met the El Escorial diagnostic criteria (1) for definite, probable or laboratory-supported probable ALS in a prospective recruitment cohort (18) from April 2000 to June 2021 at Peking University Third Hospital for comparing patients with UMND ALS and classic ALS. The protocol for electrodiagnostic evaluation of ALS includes the examination of the following muscles: one bulbar muscle (typically the sternocleidomastoid), one thoracic muscle (rectus abdominis), and one muscle each from the upper and lower limbs on both sides, totaling six muscles. For the upper limbs, the muscles usually assessed are the extensor digitorum communis, first dorsal interosseous, or abductor pollicis brevis; whereas for the lower limbs, bilateral tibialis anterior or quadriceps femoris muscles are examined. The criterion for abnormality is the presence of spontaneous activity accompanied by chronic neurogenic changes. The UMND ALS was defined as clinical features dominated by pyramidal signs, including increased deep tendon reflexes, spasticity, pathologic reflexes (Babinski, Hoffmann, snout, jaw jerk), loss of abdominal reflexes in at least three anatomically regions, the LMN signs limited to one to two muscles or minor denervation on EMG in one to two muscles on the first visit (2). Data from patients who fulfilled the criteria for UMND ALS were collected with clinical manifestations and electrophysiological results. We also excluded patients with an onset age less than 25 years, family history of motor neuron disease or spastic paraplegia to minimize the potential inclusion of HSP. Classic ALS was defined by the presence of both LMN manifestations and unquestionable UMN signs, characteristics that fulfill the El Escorial criteria for clinically definite and clinically probable ALS, while not meeting the diagnostic criteria for other subtypes of ALS. A neuromuscular neurologist (D.F.) with over 30 years of ALS diagnosis experience reviewed all cases included in this cohort. The medical history and neurological examination were extracted independently by X.L. and L.C. respectively. Detailed clinical information and electrophysiological results were extracted in standardized manner for all UMND ALS patients’ medical records.

We further categorized UMND ALS patients into two subgroups. Those who exhibited postural instability were categorized as “UMND ALS plus.” We searched for keywords in patient histories such as “falls,” “repeated falls,” and “unsteady gait,” and also looked for terms like “ataxic gait,” “unsteady gait,” and “abnormal in the retropulsion test or pull test,” in neurological examinations upon initial evaluation to identify the “UMND ALS plus” subgroup. The remaining patients were designated as “pure UMND ALS.” We collected clinical characteristics, ALSFRS-R scores and follow-up data (the endpoint of follow-up was defined as invasive ventilation or death) every 3 months. The study was approved by the Ethics Committee of Peking University Third Hospital (IRB No. 00006761). All patients in our study signed informed consent for this study. The data that support the findings of this study are available from the corresponding author upon reasonable request.

### Propensity score matching

We compared the rate of functional decline of lower extremity between subgroups after balancing the baseline difference by propensity score matching. Propensity score matching was conducted between the UMND ALS, UMND ALS plus and classic ALS cohorts with the following parameters: nearest-neighbor matching, ratio = 1:2, with a caliper of 0.2, exact matching for site of disease onset (bulbar, upper extremity, lower extremity and others) and covariates of age at onset, sex, and diagnosis delay (days). Propensity score matching was conducted using the R package *MatchIt* (Version 4.3.3) (19).

### Clinical evaluation

We extracted clinical data, including sex, age of onset, site of symptom onset (bulbar, upper extremity, lower extremity or others), additional neurological symptoms or signs (e.g., cognitive impairment and parkinsonism), disease duration and deceased status, from medical records. We compared differences in clinical phenotype, disease stage (King’s Collage staging system for ALS, KCSS) (20), Delta-FRS evaluated as the first visit (48 – ALSFRS-R score)/disease duration (months), and cognitive decline (the Edinburgh Cognitive and Behavioral ALS Screen, Chinese version, ECAS) (21) between the UMND ALS and matched cohorts. Overall and lower extremity UMN involvement was measured with the Penn Upper Motor Neuron Score (PUMNS) (22). However, Ashworth spasticity score in PUMNS may be affected by the presence of muscle rigidity in some patients, particularly in UMND ALS plus. Therefore, we excluded the Ashworth scores in the PUMNS. The semiquantitative scale score in this study ranged from 0 to 24 (0–4 for the bulbar region, 0–5 for each extremity). Upper and lower extremities strength was measured using the Medical Research Council (MRC) scale, which assessed the strength of muscle groups of proximal and distal muscles for each limb (often the more severely affected ones), with a total score ranging from 0 to 40.

To reveal the impact of lower extremity function in the early disease stage, we assessed differences in UMND ALS and matched cohort for (1) ALSFRS-R total score and lower extremity score, ranging from 0–48 to 0–8, respectively; (2) time to needs assistance, defined as the time between disease onset and walking item in ALSFRS-R < 3 points, or climbing stairs item <2 points; and (3) unable to walk, defined as the time between disease onset and ALSFRS-R walking item <2 points.

### Statistical analysis

Statistical analysis was performed using SPSS for Windows, version 26.0 (SPSS Inc.). Items were tested with Kolmogorov–Smirnov statistics to define normal distribution. Mean (SD) values are reported for normally distributed items; otherwise, median (IQR) values are reported. We used a 2-sided t test to compare normally distributed variables, the chi-square test to compare categorical variables and the Mann–Whitney U test to compare non-normally distributed variables. All tests were performed at a level of significance of *p* = 0.05.

Time to death or tracheostomy since disease onset was analyzed using Kaplan–Meier plots and log-rank tests. The simultaneous effects of variables on survival were evaluated using Cox proportional hazards regression models, and hazard ratios (HRs) were reported with 95% CIs.

## Results

### Patient characteristics

A total of 2,353 ALS patients were reviewed. 211 patients were classified as having UMND ALS, accounting for 9.0% of all ALS cases. The age of onset of the 211 UMND ALS patients ranged from 26 to 84 years (mean 53.5 years). The male-to-female ratio was 1.20; 93 patients (44.1%) had upper extremity onset, 66 (31.3%) had lower extremity onset, 51 (24.2%) had bulbar onset, and 1 (0.5%) had other onset regions. Compared with overall ALS, UMND ALS had more female patients and a shorter diagnosis delay. The demographics and clinical characteristics of all patients are listed in Table 1.

**Table 1:** Demographic and clinical characteristics of the ALS patient cohort.

Characteristic	All ALS (*N* = 2,353)	UMND ALS (*N* = 211)	*p* value for UMND ALS vs. overall ALS
Male, No (%)	1,542 (65.6%)	115 (54.5%)	<0.001
Onset age, mean ± SD	52.4 ± 12.1	53.5 ± 11.0	0.180
Diagnosis delay, median (IQR), month	14.4 (8.7–26.0)	11.6 (8.1–21.1)	<0.001
Symptom onset, No (%)			0.017
Bulbar	192 (16.2%)	51 (24.2%)	
Upper extremities	599 (50.5%)	93 (44.1%)	
Lower extremities	343 (28.9%)	66 (31.3%)	

ALS, amyotrophic lateral sclerosis; ALSFRS-R, ALS Functional Rating Scale–Revised; UMND ALS, upper motor neuron-dominant ALS.

In the UMND ALS patient cohort, we identified 33 patients who had a history of falls, and 41 patients who showed abnormal gait in neurological examinations. After eliminating duplicates, a total of 59 patients were included in the UMND ALS plus subgroup, while the remaining 152 patients were classified as pure UMND ALS. The comparison of clinical characteristics of UMND ALS plus and pure UMND ALS are listed in Table 2. UMND ALS plus patients had an earlier age of onset, longer diagnosis delay, higher incidence of lower extremity onset, and more advanced disease stage than pure UMND ALS patients. UMND ALS plus also had lower ALSFRS-R scores at diagnosis (median value 41 (37–44) vs. 44 (42–47), *p* < 0.001) and faster disease progression [Delta-FRS median value 0.419 (0.218–0.715) vs. 0.325 (0.125–0.508), *p* = 0.038] comparing with pure UMND ALS. UMND ALS plus had worse muscle strength in the lower extremity and a higher PUMNS score compared to pure UMND ALS, suggesting a more prominent dysfunction of upper motor neurons. However, sex was not different between the two groups. Even after excluding patients with lower limb onset, differences remain between the groups in terms of diagnostic delay, KCSS stage, ALSFRS-R score, Delta-FRS, assessments of upper motor neuron involvement and muscle strength (Supplementary Table). These differences suggest that there are underlying mechanisms that distinguish UMND ALS plus from pure UMND ALS beyond site of onset.

**Table 2:** The comparison of clinical characteristics of UMND ALS plus and pure UMND ALS.

Characteristic	UMND ALS plus (*N* = 59)	pure UMND ALS (*N* = 152)	*p* value for UMND ALS plus vs. pure UMND ALS
Male, No (%)	31 (52.5%)	84 (55.3%)	0.722
Onset age, mean ± SD	50.5 ± 10.4	54.6 ± 11.1	0.015
Diagnosis delay, median (IQR), month	14.1 (9.2–28.1)	11.4 (7.0–18.3)	0.005
Symptom onset, No (%)			<0.001
Bulbar	7 (11.9%)	44 (28.9%)	
Upper extremities	14 (23.7%)	79 (52.0%)	
Lower extremities	38 (64.4%)	28 (18.4%)	
KCSS stage, No (%)			<0.001
Stage 1	17 (36.2%)	99 (76.2%)	
Stage 2	22 (46.8%)	21 (16.2%)	
Stage 3	7 (14.9%)	5 (3.8%)	
Overall muscle strength (MRC)	34.0 (29.8–36.5)	36.8 (34.0–38.0)	<0.001
Lower extremity strength (MRC)	16.0 (14.0–18.0)	20.0 (18.0–20.0)	<0.001
Overall PUMNS score	14.0 (11.0–17.0)	11.0 (8.5–14.0)	<0.001
Lower extremity PUMNS score	6.0 (4.0–6.0)	4.0 (4.0–5.5)	<0.001
Cognitive decline, ECAS score, median (IQR)	105.5 (72.75–116)	90.5 (74–109.25)	0.204

ALS, amyotrophic lateral sclerosis; ALSFRS-R, ALS Functional Rating Scale–Revised; ECAS, the Edinburgh Cognitive and Behavioral ALS Screen Chinese version; KCSS, King’s Collage staging system for ALS; MRC, Medical Research Council; PUMNS, Penn Upper Motor Neuron Score; UMND ALS, upper motor neuron-dominant ALS; pure UMND ALS and UMND ALS plus, UMND ALS patients with/without postural instability or repeated falls.

### Functional assessment of the lower extremities

**Figure 1:**
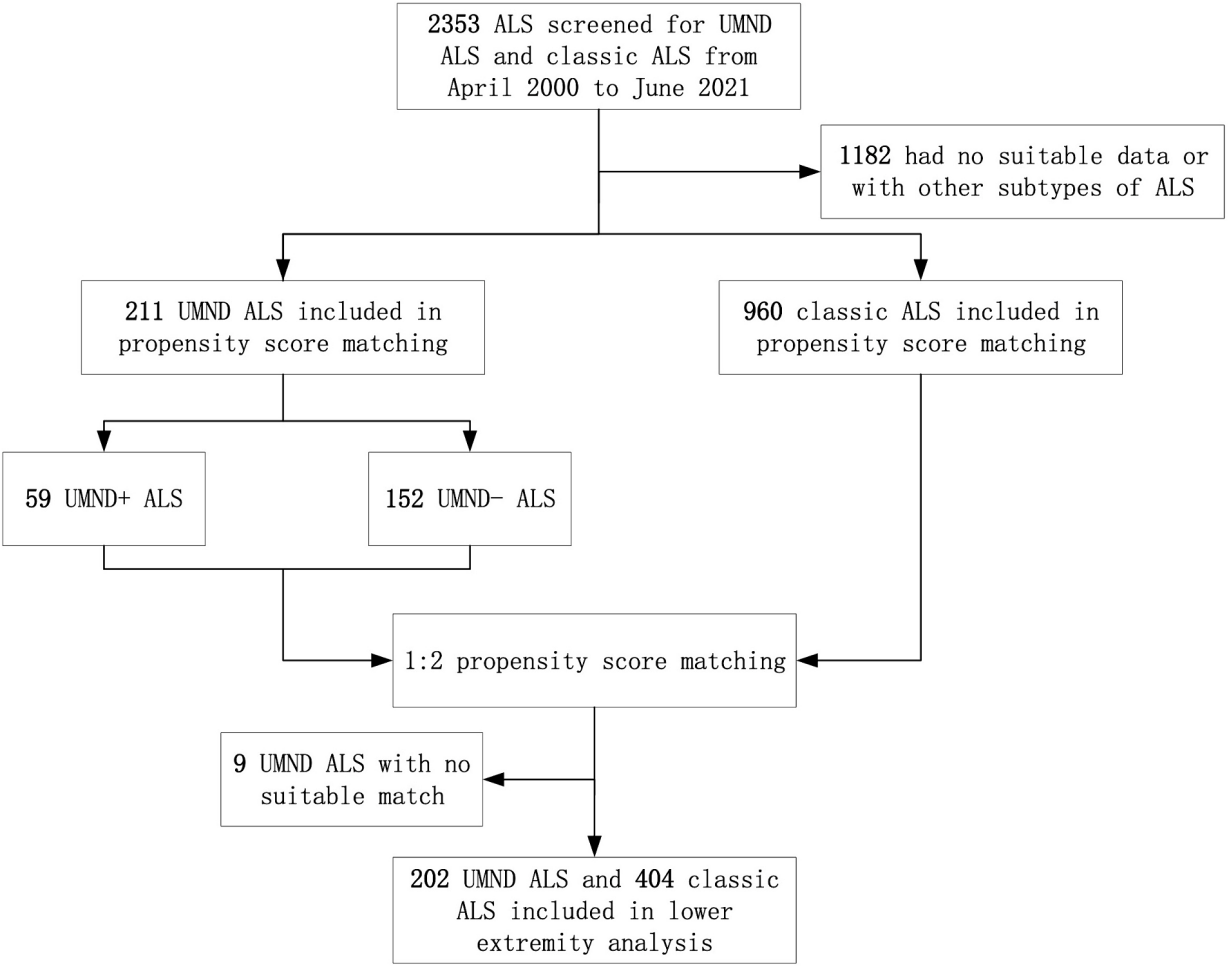
Participant flow through study analyses. ALS indicates amyotrophic lateral sclerosis; UMND ALS plus indicates upper motor neuron-dominant ALS patients with postural instability or repeated falls.

After excluding UMND ALS patients with no suitable match (9 patients), the remaining 202 patients were included in the lower extremity functional analysis (Figure 1). After propensity score matching, a total of 404 cases of classic ALS were matched for UMND ALS, and 114 cases of classic ALS were matched for UMND ALS plus cases. We further assessed lower extremity functional decline and its impact on life (Table 3). Although UMND ALS patients had better lower extremity function and strength than classic ALS patients on first evaluation, there was no difference in the time to needing assistance or not being able to walk after initial symptoms. In the UMND ALS plus subgroup, lower extremity function and strength were no better than that in the classic ALS subgroup. There was also no difference in time to lower extremity functional milestones; both UMND ALS plus and matched classic ALS patients required assistance in approximately 22 months, and unable to walk in approximately 49 months after disease onset (Table 3).

**Table 3:** Comparison of clinical characteristics and lower extremity function among UMND ALS, UMND ALS plus and matched classic ALS.

	UMND ALS (*N* = 202)	Matched classic ALS*, (*N* = 404)	*p* value for UMND ALS vs. classic ALS	UMND ALS plus (*N* = 57)	Matched classic ALS (*N* = 114)	*p* value for UMND ALS plus vs. classic ALS
ALSFRS-R at diagnosis, median (IQR), month	44 (41–46)	37 (42–44)	<0.001	41 (37–44)	42 (38–44)	0.439
ΔALSFRS-R, median (IQR), points/month	0.355 (0.144–0.590)	0.503 (0.252–0.906)	<0.001	0.441 (0.229–0.745)	0.426 (0.199–0.735)	0.845
KCSS stage, No (%)			<0.001			0.539
Stage 1	118 (68.2%)	148 (42.9%)		17 (37.0%)	47 (48.0%)	
Stage 2	41 (23.7%)	122 (35.4%)		22 (47.8%)	33 (33.7%)	
Stage 3	9 (5.2%)	35 (10.1%)		6 (13.0%)	6 (6.1%)	
Bulbar involvement	141 (69.8%)	308 (76.2%)	0.089	43 (75.4)	77 (67.5%)	0.289
Dysphagia on diagnose	56 (27.7%)	142 (35.1%)	0.066	13 (22.8%)	27 (23.7%)	0.899
Use of non-invasive ventilation on diagnose	9 (4.5%)	36 (8.9%)	0.049	1 (1.8%)	7 (6.1%)	0.202
Lower extremity strength (MRC)	18.5 (16.0–20.0)	18.0 (16.0–20.0)	0.028	16.0 (14.0–18.0)	17.0 (14.0–18.0)	0.165
Lower extremity PUMNS score	4.0 (4.0–6.0)	4.0 (0.0–5.0)	<0.001	6.0 (4.0–6.0)	4.0 (0.0–5.0)	<0.001
Lower extremity item of ALSFRS-R, median (IQR)	8 (5–8)	6 (4–8)	0.001	4 (3–5)	5 (3–6.5)	0.042
Other items of ALSFRS-R, median (IQR)	37 (34–39)	35 (31–37)	<0.001	37 (33–39)	36 (32.5–39)	0.639
Time to needing assistance, median (95% CI), month	29 (25.5–32.5)	29 (25.6–32.4)	0.729	22 (18.8–25.2)	24 (19.4–28.6)	0.153
Unable to walk, median (95% CI), month	49 (39.1–59.0)	57 (44.5–69.5)	0.949	49 (41.7–56.3)	50 (22.2–77.8)	0.630

*Propensity score matching: match for age at onset, sex, and diagnosis delay, exact match for site of disease onset (bulbar, upper extremity, lower extremity and others).

ALS, amyotrophic lateral sclerosis; ALSFRS-R, ALS Functional Rating Scale–Revised; ΔALSFRS-R, change in ALSFRS-R slope between disease onset and baseline; CI, Confidence Interval; KCSS, King’s Collage staging system for ALS; MRC, Medical Research Council; PUMNS, Penn Upper Motor Neuron Score; UMND ALS, upper motor neuron-dominant ALS; UMND ALS plus, UMND ALS patients with postural instability or repeated falls.

### Survival and multivariate analysis

**Figure 2:**
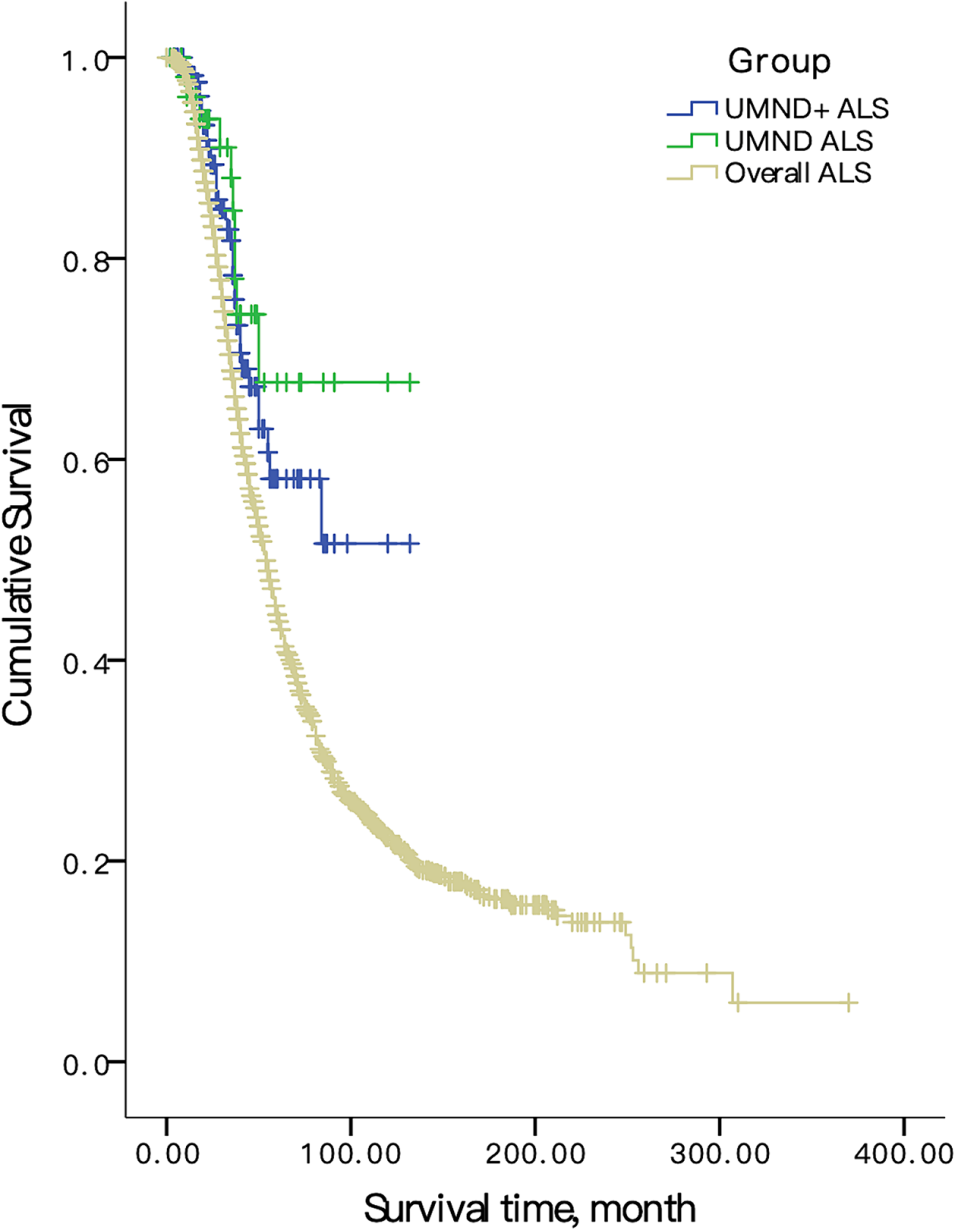
Survival curve in overall ALS, UMND ALS and UMND ALS plus. UMND ALS plus indicates upper motor neuron-dominant ALS patients with postural instability or repeated falls.

All patients in this cohort were followed-up by telephone every 3 months until January 2022. When compared with all ALS patients, the prognosis of UMND ALS and UMND ALS plus was significantly better than that of overall ALS (UMND ALS vs. overall ALS, log rank *p* = 0.002, UMND ALS plus vs. overall ALS, log rank *p* = 0.007, Figure 2). The 3-year survival probability of UMND ALS was 79.5%, and the 5-year survival probability was 56.8%. The median survival time was approximately 103 months. UMND ALS had a better prognosis than matched classical ALS (log rank *p* = 0.030).

The Cox proportional hazard regression model was used for multivariate analysis. We included age at onset, sex, diagnosis delay and site of onset as covariants in the model due to the limited number of endpoint events in UMND ALS and UMND ALS plus subgroups. The UMND ALS phenotype was an independent predictor for good prognosis after adjusting for covariant in the model (HR = 0.668, 95% CI = 0.472–0.946; *p* = 0.023). The survival time of UMND ALS plus was also better than that of classical ALS (log rank *p* = 0.032) but was not an independent predictor (HR = 0.544, 95% CI = 0.261–1.135; *p* = 0.105). There was no difference in survival for UMND ALS plus and pure UMND ALS (log rank *p* = 0.99).

## Discussion

In the present study, we examined the characteristics of UMND ALS patients and the lower extremity functional decline in a large prospective recruitment cohort. In this cohort, the prevalence of UMND ALS was 9.0%, which is comparable with previous studies. The survival of UMND ALS was significantly better than that of classical ALS, and this result was consistent after multivariate modeling. This suggests that UMND ALS is an independent predictor for good prognosis. Moreover, the patient’s KCSS stage was significantly lower than that of classic ALS, suggesting that UMND ALS has a slower progression. There was no difference in the cognitive and behavioral changes, which is also mentioned in a previous paper (7).

Although the ALSFRS-R score and lower extremity strength of UMND ALS was significantly better than that of classical ALS when the disease was diagnosed, with a generally more favorable prognosis compared to ALS, the functional decline of the lower extremities was not significantly different from that of ALS in follow-up, suggesting rapid deterioration of spasticity and instability in the early disease stage. A study also showed that the ALSFRS-R lower extremity score was numerically lower than that of classical ALS in the early stage (6). The deterioration of lower limb function may also potentially arise from the gradual development of LMN damage in UMND ALS patients, although being a less likely scenario. However, lacking follow-up EMG data, we are unable to exclude this possibility, highlighting a need for future prospective cohort study. UMND ALS patients can be included in clinical trials according to new diagnostic criteria for ALS (e.g., Gold Coast criteria) (23). According to this study, they have a nonlinear decline and “ceiling effect” of ALSFRS-R scores in the first to second year after disease diagnosis. Therefore, more attention should be paid to the mismatch between early functional decline and survival in UMND ALS, as the ALSFRS-R decline is more widely used as the surrogate outcome in clinical trials.

The current definition of UMND ALS is still empirical and debated (24). In this study, we also explored the possible heterogenicity of this ALS subtype. UMND ALS patients with gait instability or repeated falls (UMND ALS plus) account for 2.5% of all ALS patients and 28.0% of overall UMND ALS patients. UMND ALS plus has an earlier onset age, higher incidence of lower extremity onset, a lower ALSFRS-R score and a more advanced disease stage than pure UMND ALS. KCSS staging and ALSFRS-R score in patients with UMND ALS plus do not differ from those in matched classic ALS, suggesting a more rapid progression of disease that differs from pure UMND ALS. However, the lower motor neuron damage in this phenotype is relatively mild, resulting in a significantly better prognosis. The rapid decline in ALSFRS-R in UMND ALS can be attributed mainly to the UMND ALS plus subgroup, which has more severe lower extremity dysfunction. The difference on age and site of onset between UMND ALS plus and pure UMND ALS cannot be explained by differences on UMN signs or different disease stages. In addition, these two groups are stratified based on the presence of gait disturbances at diagnosis, indicating an early feature of the disease, with some patients even presenting with falls as their chief complaint. This suggests that the differences are not merely reflections of different phases of the same disease but rather implying distinct disease behaviors. Whether UMND ALS plus represents a distinct subgroup of ALS patients deserves further investigation.

The present study also showed that UMND ALS plus had a more severe (higher PUMNS score) and widespread (higher KCSS stages) UMN symptoms, consistent with a previous study (17), suggesting a more severe involvement of primary motor cortex against pure UMND ALS. However, we cannot rule out the possibility of coincidental extrapyramidal symptoms that may also contribute to lower limb dysfunction. Postural instability or backward falls have been reported in early-stage ALS, especially in UMND ALS and PLS. The premotor cortex (supplementary motor area), superior parietal lobule, and basal ganglia involvement have been speculated to be responsible for gait disorders (12, 25). Extrapyramidal features are not typical of ALS. Notably, a subset of tauopathies can also present with prominent UMN symptoms with concomitant extrapyramidal manifestations (26), similar to the UMND ALS plus in this study. Globular glial tauopathy (GGT) is a rare 4-repeat tauopathy with clinical manifestations that can mimic ALS (27). TDP-43 pathology with comorbid Tau pathology, on the other hand, is not uncommon in neurodegenerative diseases and may affect clinical manifestations. Whether Tau pathology accounts for extrapyramidal manifestations in UMND ALS plus patients still require further pathology and follow-up study. Therefore, we speculate that UMND ALS plus has widespread pathology in the brain, and the balance disturbance may be caused by a combination of severe upper motor neuron damage and extensive extra-motor brain region involvement. This hypothesis needs further pathological and neuroimaging study.

The main limitation of this study is the absence of prospective clinical follow-up, especially the assessment for lower motor neuron. Future longitudinal studies on UMND ALS would have better clarified the reason for the apparent faster progression and more significant functional decline in the UMND ALS plus subgroup. Another limitation is its baseline parameter imbalance. Although propensity score matching eliminated some of the imbalance, it also reduced representation for this cohort. During the recruitment course of this cohort, the diagnostic criteria for PLS was updated. Nonetheless, the ALS patients included in our research presented with acute and chronic denervation in at least one muscle, which still does not meet the revised diagnostic criteria for PLS (28). Given the extensive duration of the study, there is a possibility of bias in the electrophysiological data. Moreover, differentiating early-stage PLS from UMND ALS remains inherently difficult. While we cannot exclude the possibility that a minority of UMND ALS cases in this study might ultimately be diagnosed as PLS, this scenario is considered unlikely, as the UMND ALS proportion in our study aligns with prior cohorts and exhibits poorer survival outcomes compared to typical PLS. Additional limitation is that the recognition of balance disorder and gait instability in this study is based on the medical history and neurological examination, and there is no objective evaluation of balance (such as the Berg balance scale). Hence, the prevalence of UMND ALS plus patients may be underestimated. Neuroimaging, follow-up EMG, genetic and biomarker profiles were also unavailable in the current cohort and this could be clarified in future prospective cohorts. Hence, the pathophysiological mechanisms of UMND ALS cannot be addressed in this study.

In summary, UMND ALS accounts for 9.0% of all ALS patients with a favorable prognosis. However, these patients have a rapid decline in lower extremity function in the early disease stage. A subgroup of these patients with frequent falls or postural instability has an earlier disease onset and their motor function declines as fast as classic ALS. Whether these patients represent a distinct subgroup of ALS deserves further investigation.

## Data availability statement

The raw data supporting the conclusions of this article will be made available by the authors, without undue reservation.

## Ethics statement

The studies involving humans were approved by the Ethics Committee of Peking University Third Hospital (IRB No. 00006761). The studies were conducted in accordance with the local legislation and institutional requirements. The participants provided their written informed consent to participate in this study.

## Author contributions

XYL: Writing – original draft, Funding acquisition, Formal analysis. LC: Writing – review & editing, Methodology, Data curation. SY: Writing – review & editing, Formal analysis. XXL: Writing – review & editing. YZ: Writing – review & editing. DF: Writing – review & editing, Supervision, Methodology, Funding acquisition, Conceptualization.
