# Supplementary material for: Postural instability and lower extremity dysfunction in upper motor neuron-dominant amyotrophic lateral sclerosis
Source: Front Neurol. 2024 Jul 15;15:1406109. doi: 10.3389/fneur.2024.1406109 (PMC11284044; doi:10.3389/fneur.2024.1406109)
Supplement: Supplementary file 1 [file Table_1.docx]

**Postural instability and lower extremity dysfunction in upper motor neuron-dominant amyotrophic lateral sclerosis**

**Supplementary Material**

**Supplementary Table**. The comparison of clinical characteristics of UMND ALS plus and pure UMND ALS (excluding lower-limb onset patients)

| Characteristic | non-lower limb onset UMND ALS plus (N=21) | non-lower limb onset pure UMND ALS (N=124) | *P* value |
| --- | --- | --- | --- |
| Male, No.(%) | 13(61.9%) | 66(53.2%) | 0.462 |
| Onset age, mean ± SD | 50.3±11.8 | 55.0±11.1 | 0.078 |
| Diagnosis delay, median (IQR), month | 502(288.5, 964.5) | 344.5(212.25, 562) | 0.023 |
| Symptom onset, No.(%) |  |  | 0.887 |
| Bulbar | 7(33.3%) | 44(35.5%) |  |
| Upper extremities | 14(66.7%) | 79(63.7%) |  |
| Other | 0 | 1(0.8%) |  |
| KCSS stage, No.(%) |  |  | <0.001 |
| Stage 1 | 1(4.8%) | 86(69.4%) |  |
| Stage 2 | 11(52.4%) | 13(10.5%) |  |
| Stage 3 | 5(23.8%) | 3(2.4%) |  |
| ALSFRS-R at diagnosis, median (IQR), month | 37(32-41) | 45(42-47) | 0.001 |
| ΔALSFRS-R, median (IQR), points/month | 0.54(0.37-1.00) | 0.29(0.12-0.55) | <0.001 |
| Overall muscle strength(MRC) | 33.5(27.5-35.6) | 36.0(34.0-38.0) | 0.001 |
| Lower extremity strength(MRC) | 16.0(14.0-18.0) | 20.0(20.0-20.0) | <0.001 |
| Overall PUMNS score | 15.0(14.0-19.0) | 11.0 (9.0-14.0) | <0.001 |
| Lower extremity PUMNS score | 6.0(4.5-6.8) | 4.0 (4.0-5.8) | <0.001 |
| Cognitive decline, ECAS score, median (IQR) | 105(89, 118) | 88.5(74, 105) | 0.043* |

*The cut-off value of ECAS score is 82.
